# Insights from Metabolomics Profiling of MSUD in Pediatrics Toward Disease Progression

**DOI:** 10.3390/metabo15100658

**Published:** 2025-10-04

**Authors:** Abeer Z. Alotaibi, Reem H. AlMalki, Rajaa Sebaa, Maha Al Mogren, Mohammad Alanazi, Khalid M. Sumaily, Ahmad Alodaib, Ahmed H. Mujamammi, Minnie Jacob, Essa M. Sabi, Ahmad Alfares, Anas M. Abdel Rahman

**Affiliations:** 1 Department of Biochemistry, College of Science, King Saud University, Riyadh 11652, Saudi Arabia; 441203289@student.ksu.edu.sa (A.Z.A.); msanazi@ksu.edu.sa (M.A.); 2 Metabolomics Section, Precision Medicine Laboratory Department, Genomic Medicine Center of Excellence, King Faisal Specialist Hospital and Research Centre (KFSHRC), Riyadh 11211, Saudi Arabia; mmogren@kfshrc.edu.sa (M.A.M.); aalodaib@kfshrc.edu.sa (A.A.); minnie@kfshrc.edu.sa (M.J.); aalfares@kfshrc.edu.sa (A.A.); 3 Department of Medical Laboratories, College of Applied Medical Sciences, Shaqra University, Shaqra 11961, Saudi Arabia; r.sebaa@su.edu.sa; 4 Clinical Biochemistry Unit, Pathology Department, College of Medicine, King Saud University, Riyadh 11461, Saudi Arabia; ksumaily@ksu.edu.sa (K.M.S.); amujamammi@ksu.edu.sa (A.H.M.); esabi@ksu.edu.sa (E.M.S.); 5 Department of Biochemistry and Molecular Medicine, College of Medicine, AlFaisal University, Riyadh 12846, Saudi Arabia

**Keywords:** maple syrup urine disease, neonates, children, dried blood spots, untargeted metabolomics, metabolic profiling, biomarkers, physiological and external factors

## Abstract

Background: Maple syrup urine disease (MSUD) is a genetic disorder caused by mutations in the branched-chain α-ketoacid dehydrogenase (BCKDH) complex, leading to toxic buildup of branched-chain amino acids (BCAAs) and their ketoacid derivatives. While newborn screening (NBS) and molecular testing are standard diagnostic tools, they face challenges such as delayed results and false positives. Untargeted metabolomics has emerged as a complementary approach, offering comprehensive metabolic profiling and potential for novel biomarker discovery. We previously applied untargeted metabolomics to neonates with MSUD, identifying distinct metabolic signatures. Objective: This follow-up study investigates metabolic changes and biomarkers in pediatric MSUD patients and explores shared dysregulated metabolites between neonatal and pediatric MSUD. Methods: Dried blood spot (DBS) samples from pediatric MSUD patients (n = 14) and matched healthy controls (n = 14) were analyzed using LC/MS-based untargeted metabolomics. Results: In pediatric MSUD, 3716 metabolites were upregulated and 4038 downregulated relative to controls. Among 1080 dysregulated endogenous metabolites, notable biomarkers included uric acid, hypoxanthine, and bilirubin diglucuronide. Affected pathways included sphingolipid, glycerophospholipid, purine, pyrimidine, nicotinate, and nicotinamide metabolism, and steroid hormone biosynthesis. Seventy-two metabolites overlapped with neonatal MSUD cases, some exhibiting inverse trends between age groups. Conclusion: Untargeted metabolomics reveals that the metabolic profiling of MCUD pediatric patients different from that of their controls. Also, there are valuable age-specific and shared metabolic alterations in MSUD, enhancing the understanding of disease progression in MSUD patients. This supports its utility in improving diagnostic precision and developing personalized treatment strategies across developmental stages.

## 1. Introduction

Clinically, maple syrup urine disease (MSUD) is one of the inborn errors of metabolism (IEM), resulting from mutations in genes encoding the mitochondrial multienzyme complex called branched-chain α-ketoacid dehydrogenase (BCKDH) [1]. The last-mentioned enzyme, involved in the oxidative decarboxylation of branched-chain amino acids (BCAAs), including leucine, isoleucine, and valine, enables their endogenous metabolism in the body. When BCKDH is genetically mutated in MSUD, it becomes dysfunctional, leading to toxic accumulations of BCAAs and their derived ketoacids in the bodies of MSUD patients. These accumulations cause neurological damage and other serious complications, and they could be fatal if untreated [2].

Importantly, the early diagnosis and proper treatment play crucial roles in the progression and management of MSUD. MSUD patients are prescribed low-protein restricted diets because of their inability to properly metabolize the essential BCAAs and ensure adequate nutrition without triggering toxicity [3]. Sometimes, the strict diet treatment for MSUD patients is not sufficient, which leaves liver transplantation as the only option. The treatment would not be initiated until the diagnosis is confirmed [4]. For MSUD diagnosis, there are two traditional tools used to diagnose MSUD, which are newborn screening (NBS) and genetic testing. Briefly, in NBS, MSUD-selective analytes, including total leucine (Xle), isoleucine (Ile), valine, and alloisoleucine (Allo-Ile), are measured in dried blood spot (DBS) samples through tandem mass spectrometry (MS/MS) [5]. Then, positive results are molecularly confirmed by identifying genetic mutations associated with MSUD [6]. These techniques, NBS and molecular testing, have advanced the screening for MSUD with very high positive predictive value and accuracy. Still, in certain cases of MSUD with false-positive newborn screening or variants of uncertain significance (VUS), they showed certain limitations that have been of concern, necessitating consideration for improvement and prevention of inaccurate diagnosis of IEM, including MSUD [5,7,8,9]. For these reasons, clinical attempts have been made to develop an additional technique beyond traditional tools to enhance the diagnosis of IEM, improve the accuracy and reliability of NBS and molecular testing, particularly through metabolomics applications [10,11,12].

Although untargeted metabolomics has been utilized in research-oriented aspects across various fields, it has been recently proposed that untargeted metabolomics could serve as a promising additional diagnostic technique for MSUD due to its powerful analytical potential [13]. Furthermore, other studies have strongly advocated for a multi-omics approach to integrate metabolomics, genomics, transcriptomics, proteomics, and epigenomics to achieve precision diagnosis for IEM, including MSUD [14,15,16,17,18].

Technically, untargeted metabolomics comprehensively measures a wide range of metabolites at the system-level involved in various biological functions. Metabolites are very dynamic molecules produced within metabolic pathways. They act as real-time indicators of cellular activity, reflecting the physiological state of a system, and are influenced by genetic, environmental, and pathological factors or disorders [19,20].

Countably, metabolomics studies on MSUD have been performed to advance our understanding of the disease by comprehensively investigating metabolic disruptions associated with it, uncovering both primary and secondary underlying mechanisms of its pathogenesis, and discovering potential novel biomarkers for MSUD diagnosis. To date, the existing metabolomics studies are limited in number and have been conducted from varying perspectives and scopes. Specifically, they explored the pathogenesis of MSUD in terms of treatment options, including dietary management and liver transplantation, as well as the severity of the disease. Furthermore, they enhanced the diagnostic accuracy of MSUD patients with the traditional methods, as detailed below.

For instance, a comparative metabolomics study was conducted to investigate the impact of improved new diet treatment on MSUD-associated metabolic disruptions. Adolescent MSUD female patients aged 15 years without a liver transplant were age-matched with healthy controls in the study. They were given a controlled, improved medical diet for five days, followed by plasma collection and analysis through metabolomics. MSUD patients have experienced noticeable differences in lipid metabolites compared to the controls, potentially related to changes in plasma BCAAs [15]. Another study has worked on enhancing the accurate diagnosis of patients with various MSUD subtypes through their biomarkers in NBS, including BCAAs (particularly alloisoleucine). An ultra-performance liquid chromatography-tandem mass spectrometry (UPLC-MS/MS) method was developed for the accurate quantification of alloisoleucine and branched-chain amino acids (BCAAs) in DBS from classic or mild MSUD. They found that alloisoleucine was detected in all DBS from all classic MSUD patients but only in a few mild cases, indicating MSUD subtype-selective biomarkers for MSUD [21]. Furthermore, another recent study was conducted to integrate multi-omics analyses, including metabolomics, genomics, epigenomics, and transcriptomics data from fibroblasts obtained from MSUD patients and unaffected controls, aiming to precisely characterize MSUD cases and gain deeper insights into the disease’s underlying mechanisms. Their results uncovered novel epigenetic and transcriptional mechanisms associated with metabolomics underlying MSUD, including the involvement of AP1/CEBPB transcription factors and the candidate gene MEIS1 [17].

In line with this growing research, we have previously reported a disrupted metabolic profile in MSUD patients during the neonatal period. Additionally, distinct neonatal MSUD-related biomarkers were identified, which may facilitate early diagnosis. Briefly, untargeted metabolomics analyses were conducted on dried blood spot (DBS) samples taken from newborns diagnosed with MSUD and their matched healthy controls. Our promising findings indicated that MSUD newborns had affected pathways, including ascorbate and aldarate metabolism, as well as pentose and glucuronate interconversion pathways. Most importantly, the novel potential biomarkers for neonatal MSUD were L-alloisoleucine, methionine sulfoxide, and lysoPI (16:0/0:0) [14].

It is expected that the metabolic profiling of neonatal MSUD patients could evolve as MSUD patients become older, reflecting developmental and pathological changes and exposure to other external factors such as diets or treatments within the disease, possibly leading to metabolic shifts that can influence MSUD biomarkers and diagnostic reliability. Thus, it is highly recommended to first identify the metabolic profiling of pediatric MSUD patients compared to healthy controls to characterize the overall metabolic profile of the disease at this stage, considering all other factors. Then, comparative metabolomics studies would be recommended to be performed between different groups of MSUD patients with distinct ages, particularly newborns and children. Therefore, in this follow-up study, we sought to expand our prior metabolomics work in neonatal MSUD patients by incorporating a different cohort of patients from the pediatric stage to capture any age-related metabolic differences within the disease context.

## 2. Materials and Methods

### 2.1. Chemical Reagents

LC-MS-grade acetonitrile (ACN), methanol (MeOH), formic acid, and deionized water (dH_2_O) were purchased from Fisher Scientific ^®^ Company (Ottawa, ON, Canada).

### 2.2. Ethical Approval

This research adhered to the principles outlined in the Declaration of Helsinki. It received approval from the Ethics Review Board at King Faisal Specialist Hospital and Research Centre (KFSHRC) in Riyadh, Saudi Arabia (RAC# 2160 027). The approval authorized the biochemical analysis of leftover DBS cards, which does not necessitate additional consent.

### 2.3. Study Participants and Sample Collection

Twenty-eight DBS samples from study participants were obtained from the Metabolomics Laboratory, Department of Precision Medicine, Genome Medicine Center of Excellence, at KFSHRC. The study included fourteen children with biochemically and genetically confirmed MSUD and fourteen age- and gender-matched healthy controls. Participants aged 8 to 13 years with a confirmed MSUD diagnosis and their matched controls were enrolled. MSUD patients over 13 years old or affected by other IEMs were excluded based on the study criteria. The specific age was selected as in many clinical and research settings, age 13 years and above is treated differently from younger pediatric age groups for several important reasons, including reducing variability due to puberty, ensuring consistency in disease stage or presentation, and making results easy to interpret for a specific and defined developmental stage. Blood samples were collected, spotted onto Whatman 903 Protein Saver cards, dried, and stored at 4 °C. These DBS samples were then analyzed using NBS with tandem mass spectrometry and the CE-marked MassChrom kit (ChromSystems, Gräfelfing, Germany), which detects MSUD-related targeted markers, such as isoleucine and valine.

### 2.4. Metabolite Extraction

Metabolites were extracted from DBS using a standard procedure previously published [21]. Briefly, metabolites were extracted from each DBS card using a 3.2 mm punch, which was incubated with 250 μL of an extraction solvent composed of deionized water, methanol, and acetonitrile (1:2:2). The mixture was vortexed with a ThermoMixer (Eppendorf, Hamburg, Germany) for 2 h at 600 rpm and 25 °C. After incubation, the samples were centrifuged at 16,000 rpm for 10 min at 4 °C. The resulting supernatants were then dried using a SpeedVac concentrator (Thermo Scientific, Dreieich, Germany; Christ, Munich, Germany). Notably, another set of punches from each sample was collected to prepare pooled QC samples to monitor the system’s stability.

### 2.5. LC-HRMS Metabolomics Analysis

The extracted samples were reconstituted with 50% mobile phase A (0.1% formic acid in dH_2_O) and 50% mobile phase B (0.1% formic acid in a 1:1 *v*/*v* mixture of MeOH and ACN). A 5 μL sample was injected into the ACQUITY UPLC XSelect C18 (100 × 2.1 mm × 2.5 μm) column (Waters Ltd. in Elstree, UK). The mobile phase flow rate was 300 μL/min, with the column maintained at 55 °C. In gradient mode, mobile phases A and B were pumped through the column, starting with 95% A for 16 min, followed by 5% A held for 4 min, then transitioning to 95% A over 2 min, and held at 95% A for another 2 min. All phases were kept at 4 °C, and the LC system was equipped with an electrospray ionization (ESI) source for molecule separation, connected to an Xevo G2-S QTOF mass spectrometer (Waters Ltd., Elstree, UK). Metabolites were ionized at 150 °C. The capillary voltage was 3.20 kV in ESI+ mode and 3 kV in ESI− mode. The cone gas flow was 50 L/h, and the gas flow was 800 L/h. The cone voltage was set at 40 V. In MSE DIA mode, collision energies ranged from 5 to 10 eV. The MS was calibrated with sodium formate (100–1200 Da) in both ionization modes. The lock spray mass compound was MS leucine-enkephaline (an external reference at *m*/*z* 556.2771 in positive mode and 554.2615 in negative mode). The scan time was 0.5 s, with a flow rate of 10 μL/min, and cone collision energies of 4 and 30 V. DIA data were collected using Waters Inc.’s MassLynx™ V4.1 software (Milford, MA, USA). Quality control (QC) samples were used to monitor system stability, as detailed in [21], with pooled QC results showing a relative standard deviation (RSD) of less than 40% [22].

### 2.6. Metabolomics Data Processing, Annotation, and Statistical Analysis

The MS raw data were processed using a standard protocol, which aligned ion signals based on mass-to-charge ratio (*m*/*z*) and retention time (RT), followed by peak detection and annotation of the features through Progenesis QI v.3.0 software (Waters Technologies, Milford, MA, USA). The chemical structures of metabolites were determined through the Human Metabolome Database (HMDB) [23]. The tolerance for hypothesized tandem MS was 5 ppm. Exogenous metabolites were removed from the final list.

Statistical analysis of pediatric metabolomics data was conducted using MetaboAnalyst v.5.0 (McGill University, Montreal, QC, Canada; https://www.metaboanalyst.ca/), accessed on 12 January 2024. The imported data undergo median normalization, Pareto scaling, and log transformation. Normalized data were used to create partial and orthogonal partial least squares discriminant analysis (PLS and OPLS-DA). The OPLS-DA model’s effectiveness was evaluated using R^2^Y and Q^2^ [24]. A univariate analysis was performed using Mass Profiler Professional (MPP) software v.15.0 (Agilent, Santa Clara, CA, USA). Using a moderated *t*-test (cut-off: FDR *p* value less than 0.05, fold change 2). A volcano plot was generated to identify mass features. MetaboAnalyst v5.0 for biomarker analysis generated a receiver operating characteristic curve (ROC) and pathway analysis. The Area Under Curve (AUC) determines sensitivity, specificity, and accuracy. MPP software (Agilent Inc., Santa Clara, CA, USA) generated a Venn diagram to identify common metabolites in patients with MSUD. For comparative statistical analysis, the neonatal metabolomics data from our previously published work [13] were reanalyzed alongside the pediatric metabolomics data generated in this follow-up study. Thus, the significant endogenous metabolites found in neonatal samples (n = 210) were used for the comparative analyses with the significant endogenous metabolites in pediatric samples (n = 1080). These comparative analyses aim to identify shared metabolites between neonatal and pediatric MSUD samples and to evaluate whether their levels are influenced by age or disease progression.

## 3. Results

### 3.1. Demographic and Clinical Features of Pediatric Participants

Pediatric participants were age- and gender-matched. The average age of pediatric MSUD patients was (11 ± 0.277) years, while the matched controls’ age was (10.85 ± 0.345) years. Patients over 13 years old and any individuals with IEM cases, except for MSUD, were excluded. Participants were equally divided between males and females, with 50% of each gender. According to the results of tandem mass spectrometry (MS/MS), pediatric MSUD patients had significantly higher levels of Xleucine (1003.36 ± 65.14 µM) and Valine (622.5 ± 44.63 µM) compared to the matched controls, who had normal levels of Xleucine and Valine that were (218.7 ± 4.96 µM) and (260.7 ± 4.46 µM), respectively, as shown in Table 1.

### 3.2. Untargeted Metabolomics Profile of MSUD Patients

**Figure 1:**
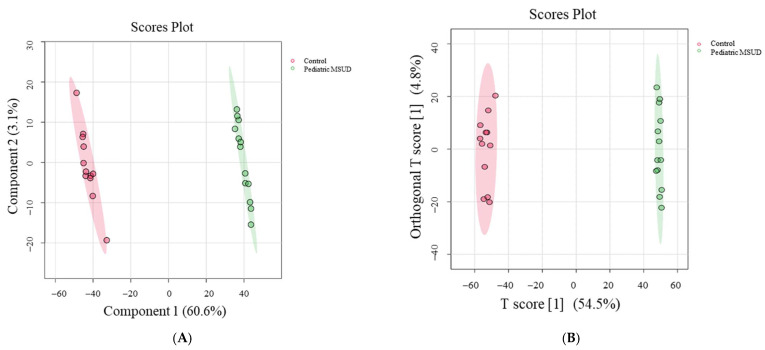
Sample clustering and group separation based on a group of 16.231 features. (**A**) PLS-DA separates 28 samples from pediatric MSUD patients (green dots) and healthy controls (pink dots). (**B**) OPLS-DA indicates the clear separation between pediatric MSUD patients and healthy control groups. The robustness of the generated models was assessed by the fitness of the model (R^2^: 0.99985) and predictive ability (Q^2^: 0.99165) values.

A total of 28,769 ion features were detected, including positive (n = 19,336) and negative (n = 9433) ionization modes. The data were deposited in the Metabolomics Workbench (ST003171). After applying a filter with a threshold of 80%, missing values (28.5%) were excluded to ensure quality, resulting in 16,231 features. To confirm that all depicted data have a Gaussian distribution, the median, log-transformation for normalizing the data, and Pareto scaling are used to remove variances. Multivariate analysis using PLS-DA revealed sample clustering and a clear separation between pediatric MSUD patients (green dots) and healthy controls (pink dots), as shown in Figure 1A. Additionally, OPLS-DA demonstrated a clear separation in the score plot, with a computed R^2^Y = 0.999 and Q^2^ = 0.991, as shown in Figure 1B.

**Figure 2:**
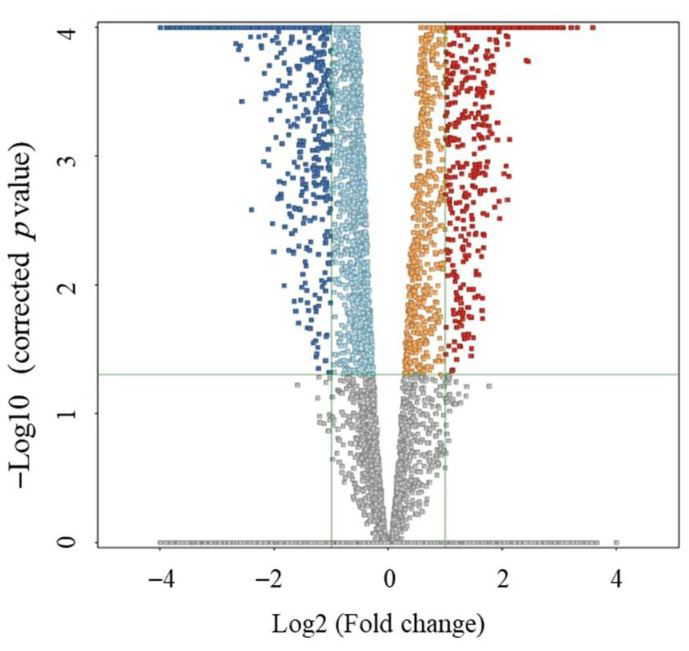
Volcano plot (Moderated T-test, cut-off: FDR, *p* ≤ 0.05, FC cut-off 2) between pediatric MSUD patients and healthy controls. It revealed 7754 significantly dysregulated metabolites, of which 3716 (reddish dots) and 4038 (blueish dots) were up- and down-regulated, respectively.

A univariate analysis was performed after normalizing the signal and ensuring that the distribution was normal. Volcano plot (Moderated Student *t*-test, cut-off: FDR, *p*-value ≤ 0.05 and fold change 2) was used to identify significantly altered features between pediatric MSUD patients and controls. The significant dysregulated metabolites, 7754 in total, where 3716 and 4038 are up- and downregulated, respectively, are shown in Figure 2. An annotation of 3432 metabolites was achieved using HMDB. Identification of metabolites resulted in 1080 endogenous metabolites, excluding the exogenous metabolites, listed in Table S1.

### 3.3. Analysis of Metabolomic Pathway

**Figure 3:**
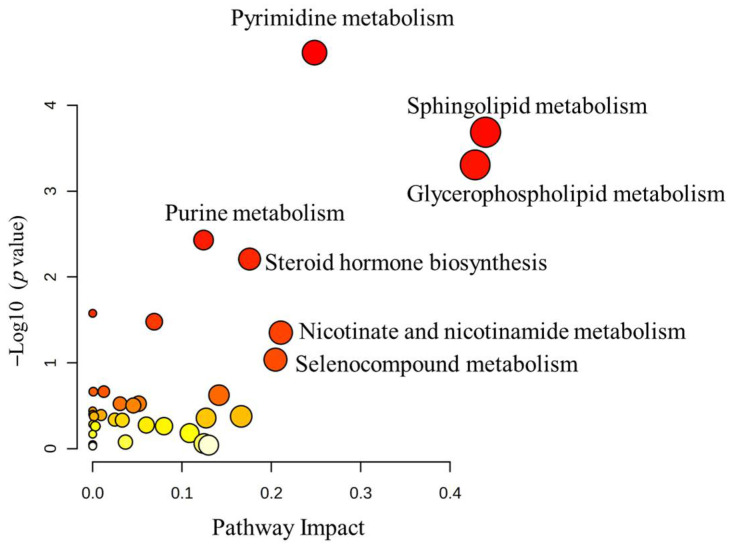
Pathway analysis of the significant dysregulated metabolites in MSUD patients. The color variation in the colors (yellow to red) indicates the metabolites with varying significance levels and their impact on the data.

All 1080 dysregulated metabolites were subjected to pathway enrichment analysis to identify the most altered pathways. Pathway enrichment analysis identified the most significantly affected metabolic pathways. The top-impacted pathways were pyrimidine metabolism, sphingolipid metabolism, glycerophospholipid metabolism, purine metabolism, steroid hormone biosynthesis, and nicotinate and nicotinamide metabolism, as shown in Figure 3 and Table S2.

### 3.4. Biomarkers Analysis of Pediatric MSUD

**Figure 4:**
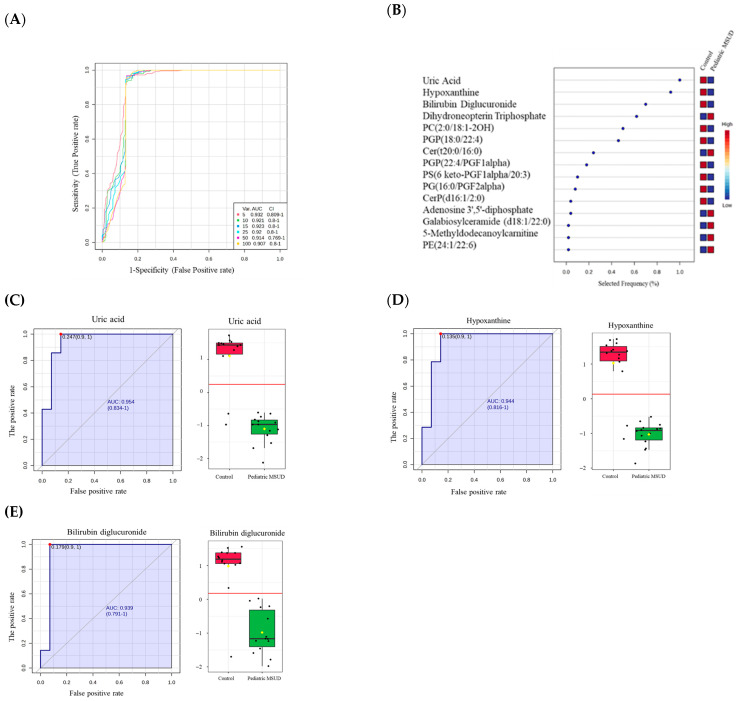
Metabolomics profiling and biomarker evaluation between pediatric MSUD and healthy groups. (**A**) The receiver operating characteristics (ROC) curve was created by the OPLS-DA model, with Area Under Curve (AUC) values calculated from the combination of 5, 10, 15, 25, 50, and 100 metabolites. (**B**) The frequency plot shows the top 15 identified metabolites. Figure (**C**–**E**) illustrates examples of dysregulated biomarkers in pediatric MSUD patients, such as uric acid (**C**), hypoxanthine (**D**), and bilirubin diglucuronide (**E**), that were downregulated with respective AUC of 0.954, 0.944, and 0.939, respectively.

To identify pediatric biomarkers, receiver operating characteristic (ROC) curves were generated for the significantly endogenous dysregulated metabolites between pediatric MSUD patients and healthy controls. Classifications and features were analyzed through the ranking approach, PLS-DA, to create a multivariate exploratory ROC analysis. Six features in the ROC curve obtained from PLS-DA and cross-validation (CV) had an area under the curve (AUC). The ROC curve showed a set of variant metabolites (5, 10, 15, 25, 50, and 100), with different AUCs and confidence intervals (CIs) (Figure 4A). The frequency plot showed the top 15 metabolic biomarkers with the highest scores, remarkably including, uric acid, hypoxanthine, bilirubin diglucuronide, dihydroneopterin triphosphate, PC(2:0/18:1-2OH(9,10)), PGP(18:0/22:4), Cer(t20:0/16:0), PGP(22:4/PGF1alpha), PS(6 keto-PGF1alpha/20:3), PG(16:0/PGF2alpha), CerP(d16:1/2:0), Adenosine 3′,5′-diphosphate, Galabiosylceramide (d18:1/22:0), and 5-Methyldodecanoylcarnitine, and PE(24:1/22:6) (Figure 4B). Examples of biomarkers were mentioned, such as uric acid, hypoxanthine, bilirubin diglucuronide, and dihydroneopterin triphosphate (Figure 4C–E).

### 3.5. Dysregulated Metabolites Shared Between Neonatal and Pediatric MSUD Patients

**Figure 5:**
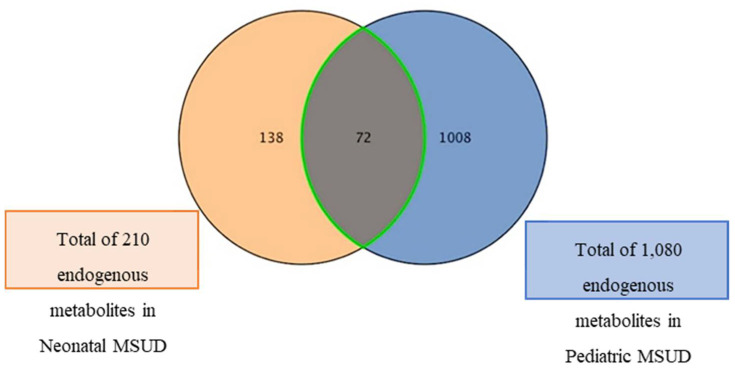
The Venn diagrams showed 72 shared dysregulated metabolites between neonatal and Pediatric MSUD patients. In comparison, 138 novel metabolites were specific to newborn MSUD (orange), and 1008 unique metabolites were specific to pediatric MSUD (blue).

We aimed to identify metabolites dysregulated in MSUD-related conditions shared between newborns and children diagnosed with MSUD, and to characterize these metabolites, including whether their levels were affected by age or disease progression. Thus, further statistical analyses were performed on the list of dysregulated metabolites in neonatal MSUD patients (n = 210), previously published by our research group [14], and on the dysregulated metabolites in pediatric MSUD patients (n = 1080). Specifically, Venn diagrams have shown that 72 metabolites were affected by MSUD detectable from the onset of disease and continuing into childhood. The overlapping of the 72 metabolites suggests their substantial association with disease progression, while the remaining metabolites are those that have changed due to exposure to external factors (Figure 5).

Interestingly, of the 72 shared metabolites, 24 metabolites were decreased and 18 metabolites were increased in pediatric MSUD compared to neonatal MSUD patients. However, 30 shared metabolites have shown no differences between neonatal and pediatric MSUD patients, persisting at a similar level of alteration from the onset of disease at the early stage until childhood (Table S3). Notably, the biomarker analysis revealed no shared metabolic biomarkers between neonatal and pediatric patients with MSUD.

## 4. Discussion

### 4.1. Untargeted Metabolomics Offers a Framework for Characterizing Metabolic Trajectories Across Developmental Stages

In particular, the human metabolic system undergoes significant rapid changes following birth to adapt to the required physiological functions with age [25]. Various noticeable changes in the newborns’ bodies are observed as they progressively grow, including the maturation of organ function, enzymatic activity, and physiological processes. The last features can influence the systemic or tissue metabolic signature in the human body [26,27,28]. Thus, the transition from the neonatal to the pediatric period is very critical and unstable, involving various metabolic shifts. Therefore, performing comprehensive systemic metabolomics across early developmental stages, ranging from newborns to children, provides fundamental information about the systemic metabolic reactions/pathways in the body under normal developing physiological conditions, which can serve as a basis for disease studies.

Understanding the human metabolome’s response to developmental stages under normal conditions enables researchers to identify metabolome alterations in diseases across different ages or in response to clinical treatments, dietary management, or other age-related factors. Additionally, it may aid in identifying metabolic biomarkers for each developmental stage under both physiological and pathological conditions. Furthermore, it could eventually assist in developing effective treatments and prevention approaches to be applied to diseased patients at different developmental stages and ages [29,30,31,32,33].

Previously, we focused on elucidating the metabolic alterations of MSUD during the neonatal period and identifying neonatal MSUD-specific biomarkers for early diagnosis. We conducted an untargeted metabolomics analysis on DBS samples collected from neonatal MSUD patients and healthy controls, identifying significant metabolic differences in the neonatal MSUD patients compared to the controls. Neonatal MSUD-specific biomarkers were detected, including L-alloisoleucine, methionine sulfoxide, and lysoPI (16:0/0:0) [14].

Herein, we follow up on our previous metabolomic study, focusing on another developmental stage, “childhood,” and performing untargeted metabolomics analyses on DBS samples from pediatric MSUD patients to investigate metabolic alterations in MSUD that persist for years after birth. Secondly, we conducted comparative metabolomics analyses between neonatal and pediatric metabolomic profiling data to assess metabolic shifts related to developmental stage within the context of MSUD. Overall, our findings illustrated that pediatric MSUD patients had an altered metabolic profile compared to their matched healthy controls. Secondly, pediatric MSUD patients exhibited distinct metabolic biomarkers that were not shared with neonatal MSUD patients, suggesting the age- and development-specificity of MSUD biomarkers. Lastly, pediatric and neonatal MSUD shared common dysregulated metabolites, but the direction and expression of several shared metabolites were inversely regulated between the two groups. In brief, our findings revealed that pediatric MSUD patients exhibited distinct metabolic alterations compared to healthy controls. Additionally, our results demonstrated that each developmental stage (neonates and/or children) exhibited distinct and dynamic metabolic biomarkers that could be considered for diagnosis and management, either at earlier or later stages of development.

### 4.2. Different Metabolomics Patterns Between Pediatric MSUD Patients and Healthy Controls

Our untargeted metabolomics findings indicated that pediatric MSUD patients had significant metabolic alterations at the systemic level, observed in several metabolic pathways and their metabolites. In comparison with healthy controls, pediatric MSUD patients had impacted pathways including pyrimidine metabolism, sphingolipid metabolism, glycerophospholipid metabolism, purine metabolism, steroid hormone biosynthesis, and nicotinate and nicotinamide metabolism. Although MSUD is a disease associated with the disrupted metabolism of BCAAs [1], the affected pathways in MSUD patients extend beyond BCAA metabolism and involve broader systemic metabolic disturbances. One of the possibilities is that the accumulations of BCAAs in MSUD patients could trigger indirectly other interconnected metabolic pathways, possibly cause secondary effects, and contribute to the pathogenesis of the disease. For instance, our findings revealed that many metabolites involved in sphingolipid and glycerophospholipid metabolism were altered, including sphingomyelin, n-acylsphingosine, digalactosylceramide, glucosylceramide, d-glucosylsphingosine, GM3, GA2, phosphatidylcholine, 1-acyl-sn-glycero-3-phosphocholine, choline, phosphatidate, CDP-ethanolamine, CDP-glycerol, and phosphatidylglycerol. They are lipid species involved in cell membranes, lipid signaling, and energy metabolism. Alterations in their levels suggest that the MSUD condition may induce oxidative stress events and changes in complex lipid metabolism, leading to cellular membrane damage or altered lipid signaling in the brain or other tissues. The last possibility is supported by other previous studies on other neurodevelopmental diseases [34,35]. These alterations in these metabolites could cause disrupted myelin formation and severe neurotological impairments. Furthermore, it has been reported that the accumulation of branched-chain α-keto acids in maple syrup urine disease causes morphological alterations and oxidative stress in glial cells [36]. In another study, they found that oxidized phosphatidylcholines have been proposed as novel biomarkers of oxidative stress in neurological conditions [34].

In addition, metabolites involved in nicotinate and nicotinamide metabolism were affected in MSUD patients, as seen in deamino-NAD^+^, nicotinate D-ribonucleoside, and NADP. These metabolites are fundamental for maintaining cellular redox balance, energy metabolism, and cofactor biosynthesis [37,38]. These alterations result from the mitochondrial impairment and oxidative stress in MSUD and act as a compensation mechanism to maintain redox homeostasis and energy balance, potentially causing fluctuations in the levels of NAD^+^, NADP^+^, and their associated precursors or analogs.

In our current study, purine and pyrimidine metabolism were secondarily impacted in pediatric MSUD patients. In agreement with our current metabolomics findings, studies showed that MSUD patients have disrupted purine and pyrimidine metabolism. Specifically, they observed abnormalities in key purine metabolites, including hypoxanthine, inosine, and xanthosine, as well as in pyrimidine metabolites such as uridine, cytidine, and dihydrothymidine. These metabolic disruptions suggest that nucleotide metabolism is indirectly affected, most probably as a downstream consequence of BCAA accumulation, mitochondrial dysfunction, and redox imbalance characteristic of MSUD pathology [14,16].

Last but not least, steroid metabolism was perturbed in MSUD cases, suggesting the secondary impact of BCAA on steroid metabolism-related metabolites. Another recent study revealed an indirect link between MSUD and steroid metabolism. This was demonstrated through a single case study involving a patient with MSUD who was given an improved restricted diet for five days, followed by metabolomics analysis of a blood sample taken from the patient. Their results suggested a potential connection between BCAA imbalance and disrupted steroid metabolism [15]. Collectively, our metabolomics results demonstrate that MSUD pathophysiology consists of complex systemic metabolic alterations resulting from either the direct impact of BCAAs accumulation or the indirect consequences of disruptions to interconnected metabolic processes.

### 4.3. Distinct Biomarkers of Pediatric MSUD Compared to Neonatal MSUD

Surprisingly, our metabolomics analysis revealed metabolic biomarkers in pediatric MSUD patients that differed from those identified in our previous study on neonatal MSUD patients [14]. In this current study, we found that pediatric MSUD patients had uric acid, hypoxanthine, and bilirubin diglucuronide as biomarkers. Hypoxanthine and uric acid are known to be involved in purine metabolism, acting as important intermediates/products in nucleotide regulation. Metabolically, hypoxanthine in purine metabolism is converted to xanthine, which is then further metabolized to uric acid as the final product [39,40,41]. Interestingly, there is no direct link between the two biomarkers and BCAAs metabolism; however, the biomarker results suggest the possibility of system-wide effects of BCAAs on purine metabolism-related biomarkers. In agreement with our findings, it has recently been reported that uric acid is a biomarker for acute metabolic decompensation (AMD) in pediatric MSUD patients [42].

Additionally, bilirubin diglucuronide was identified as a potential biomarker in pediatric patients with MSUD. It was decreased in MSUD patients compared to healthy controls. Bilirubin diglucuronide is a water-soluble metabolite produced in the liver by the action of an enzyme called UDP-glucuronosyltransferase. It is needed for the detoxification and elimination of catabolic products of heme molecules [43]. Since the liver could be dysfunctional in the MSUD condition, and some MSUD patients have to receive a transplanted liver as a treatment [4], it could be explained that the decreased level of bilirubin diglucuronide in pediatric patients is due to the functional impairment in the liver in MSUD function, which consequently affects the production of the liver byproduct metabolites (bilirubin diglucuronide) as a secondary effect.

Notably, the liver is a vital organ that performs multiple functions, including the production of uric acid, hypoxanthine, and bilirubin diglucuronide, which can be easily targeted and detected in pediatric MSUD. These metabolic biomarkers may be selectively useful for pediatric MSUD; however, further validation is required to use them for diagnosis at developmental stages.

### 4.4. Inverse Expression of Dysregulated Metabolites Shared Between Neonatal and Pediatric MSUD Patients

Notably, our untargeted metabolomic analyses of MSUD samples revealed that pediatric and neonatal MSUD patients shared 72 dysregulated metabolites, which are intermediates in the metabolism of energy, glycerophospholipids, nucleotides, and sphingolipids. However, some of these metabolites were altered during developmental stages, with their levels being inversely expressed in neonates compared to children. To illustrate, the same metabolites were increased in neonates but decreased in children with MSUD (or vice versa), suggesting that age-specific metabolic changes occur. These changes may be attributed to various physiological or external factors, including organ maturation, cellular adaptations, enzymatic activity, and regulatory mechanisms [44,45]. These factors could influence the body’s handling of these metabolites in the context of MSUD.

Physiologically, the neonatal period is characterized by the most dramatic physiologic changes that occur during human life. Postnatally, certain organs and tissues mature through different developmental stages, and numerous alterations in metabolites can occur during this maturation process. For instance, immature clearance mechanisms in babies result in increased levels of toxic metabolites, but these levels drop in children as their systems mature [46,47,48,49]. Thus, it is essential to note that the inverse metabolites between neonates and children are not solely due to MSUD, but also to the natural organ/tissue maturation and development process that occurs in newborns after birth, which should be taken into consideration. Then, MSUD neonates may develop compensatory mechanisms to overcome the overlapping defects that arise in the context of the disease as they grow to survive.

One possible explanation for the inverse metabolite patterns is that newborns diagnosed with MSUD receive restricted diets and/or therapeutic interventions early on. Continued intervention through childhood may alter the metabolic fingerprint, leading to changes in shared metabolites. During the neonatal period, the major energy source is ketone bodies, typically derived from the high-fat content of breast milk. Neonatal tissues/organs, especially the brain, utilize ketone bodies to meet their energy requirements. As newborns develop, their preferred metabolic pathways shift from ketone body metabolism to carbohydrate metabolism. This metabolic shift is attributed to developmental changes, liver maturation, hormonal regulation, and the consumption of increased carbohydrate-containing foods. By early childhood, glucose becomes the dominant energy source [50,51]. In the case of MSUD, these normal metabolic shifts induced by developmental stages can be negatively impacted, contributing to the inverse expression of shared metabolites observed between neonates and older children.

To summarize, the metabolic interplay in pediatric MSUD is more complex than in the neonatal stage. This increased metabolic complexity arises from the interaction of many physiological and external factors within the pathogenesis of MSUD. Notably, physiological factors encompass growth, hormonal changes, organ maturation, and the development of evolving enzyme activity. External factors are diet, medication, and ongoing therapeutic interventions. These factors can alter metabolic pathways over time, making metabolic profiling in pediatric MSUD patients more challenging and variable than in neonatal MSUD patients. Therefore, the shared metabolites between the two age groups may appear differently expressed, reflecting the dynamic nature of metabolic regulation as the child grows, and merit further and deeper investigation.

### 4.5. Limitations and Future Directions of the Study

Future studies are needed to address specific areas and strengthen our understanding of the metabolomics aspects of MSUD. Longitudinal metabolomics studies that follow MSUD patients from the neonatal stage through childhood are necessary to reveal age-specific biomarkers and track disease progression. Specifically, age-specific biomarkers may enhance clinical management by supporting treatment monitoring and informing dietary adjustments across various developmental stages. Secondly, it is fundamentally essential to integrate metabolomics with other omics approaches, such as genomics, transcriptomics, and proteomics, to gain a more comprehensive understanding of the underlying mechanisms and molecular basis of MSUD. Furthermore, larger cohorts of MSUD studies are crucial for validating the initial metabolomics findings. Moreover, studies exploring dietary strategies, pharmacological agents, and the involvement of the microbiome, as well as their impacts on the metabolic profile of MSUD, are valuable for gaining a broader understanding of their contributions to disease regulation. In addition, it would be of great interest to investigate altered pathways and biomarkers associated with MSUD, whether these are shared with or distinct from those of other metabolic diseases. Finally, it is worth noting that identifying metabolic biomarker-based diagnostics for MSUD patients across various developmental stages is crucial for enhancing early detection and disease management, as well as translating these findings into clinical applications.

## 5. Conclusions

Our findings highlighted that untargeted metabolomics is a valuable and powerful tool used for understanding the pathogenesis of pediatric MSUD and identifying potentially relevant biomarkers. It was evident that pediatric MSUD patients exhibited distinct metabolic profiles compared to health-matched controls. Furthermore, the biomarker profile of neonatal MSUD differs from that observed in pediatric patients. Although certain dysregulated metabolites were shared between neonates and children, their expression was inversely regulated, indicating not only the impact of MSUD itself but also the influence of developmental stage, therapeutic intervention, and disease progression. Considering all these factors in the interpretations of metabolomics analyses of MSUD, it would provide more accurate insights, ultimately supporting the development of improved, age-specific personalized treatment strategies for MSUD patients.

## Figures and Tables

**Table 1:** Demographic and Clinical features of pediatric participants.

Demographic and Clinical Features		Pediatric MSUD (n = 14)		Control (n = 14)		*p*-Value
		Mean	SEM	Mean	SEM	
**Age (years)**		11	±0.277	10.85	±0.345	0.72
**Gender**	**Female** (%)	50	NA	50	NA	NA
	**Male** (%)	50	NA	50	NA	NA
**Biomarker**	**Xleucine** (Cutoff: <245 µM)	1003.36	±65.14	218.7	±4.96	1.59 × 10^−11^ **
	**Valine** (Cutoff: <290 µM)	622.5	±44.63	260.7		4.01 × 10^−8^ **

For statistical analyses, an independent Student’s *t*-test was conducted. Mean ± SEM expresses data. Student’s *t*-test: Two-tailed was applied (** *p* < 0.01); MSUD: Maple Syrup Urine Disease; SEM: standard error of the mean; Xleucine (i.e., the total of leucine and isoleucine).

## Data Availability

The raw data of this study were deposited at the Metabolomics Workbench on 29 April 2024, and can be accessed under accession number ST003171.

## Supplementary Materials

The following supporting information can be downloaded at: https://www.mdpi.com/article/10.3390/metabo15100658/s1, Table S1: Significantly dysregulated endogenous metabolites were in pediatric MSUD patients compared to healthy controls. (Moderated *t*-test, FDR *p*-value < 0.05, Fold change 2); Table S2: Pathway analysis showed the significantly impacted pathways and their dysregulated metabolites in pediatric MSUD patients compared to healthy controls; Table S3: comparative study of shared dysregulated metabolites between pediatric and neonatal MSUD patients.
